# Extracranial Hemodynamic Responses to a Noxious Cold Pressor Task Differ Between Persistent Post-Traumatic Headache and Healthy Controls

**DOI:** 10.3390/jpm15120593

**Published:** 2025-12-03

**Authors:** Aaron W. Parr, David B. Berry, Bahar Shahidi, Dawn M. Schiehser, Katrina S. Monroe

**Affiliations:** 1 Joint Doctoral Program in Public Health, University of California San Diego, 9500 Gilman Dr., San Diego, CA 92093, USA; aparr2098@sdsu.edu; 2 Joint Doctoral Program in Public Health, San Diego State University, 5500 Campanile Dr., San Diego, CA 92182, USA; 3 Department of Orthopaedic Surgery, University of California San Diego, 9500 Gilman Dr. MC0863, La Jolla, CA 92093, USA; dbberry@health.ucsd.edu (D.B.B.); bshahidi@health.ucsd.edu (B.S.); 4 VA San Diego Healthcare System, 3350 La Jolla Village Dr., San Diego, CA 92161, USA; dschiehser@health.ucsd.edu; 5 Department of Psychiatry, University of California San Diego, 9500 Gilman Dr., San Diego, CA 92093, USA; 6 School of Physical Therapy, College of Health and Human Services, San Diego State University, 5500 Campanile Dr., San Diego, CA 92182, USA

**Keywords:** autonomic nervous system, cortical, functional neuroimaging, headache disorders, secondary, nociception, near-infrared spectroscopy

## Abstract

**Background/Objectives:** Headache after a traumatic brain injury (TBI) is one of the most common post-concussive symptoms and is associated with altered pain processing and elevated disability levels. Understanding physiologic correlates of nociception in individuals with persistent post-traumatic headache (pPTH) may help identify novel treatment targets for pain-related disability. The objective of this case–control study was to compare extra- and intracranial hemodynamic responses to a noxious cold pressor task (CPT) between individuals with pPTH and healthy controls (HC) using functional near-infrared spectroscopy (fNIRS). **Methods:** Ten individuals with pPTH were compared to ten HC with no history of TBI, persistent headache, or chronic pain. fNIRS optodes over the medial prefrontal cortex (PFC) measured extra- and intracranial peak-to-peak hemodynamic responses during tepid- (control) and cold-water (CPT) hand immersion. Evoked pain responses during the CPT were assessed with numeric pain ratings. Linear mixed effects modeling assessed the role of group and evoked pain on hemodynamic responses. **Results:** pPTH group membership (*p* = 0.031) predicted greater extracranial hemodynamic responses to the CPT, whereas intracranial PFC responses did not differ between groups. Regardless of group membership, greater increases in pain intensity during the CPT were associated with increased hemodynamic responses for the dorsomedial PFC (*p* = 0.031). **Conclusions:** Compared to controls, individuals with pPTH responded to a noxious cold stimulus with elevated systemic hemodynamic responses regulated by the autonomic nervous system. Irrespective of group, hemodynamic responses within the dmPFC were associated with evoked pain responses to the CPT and may provide a useful biomarker for individual variations in cortical pain processing for healthy and clinical populations.

## 1. Introduction

Headache after a mild or moderate traumatic brain injury (mmTBI) is one of the most common post-concussive symptoms and is related to high levels of disability [1,2]. Despite many individuals seeking treatment, an estimated 47% continue to experience persistent post-traumatic headache (pPTH) three or more months after the onset of symptoms [2]. Understanding physiologic changes related to the processing of nociceptive stimuli in individuals with pPTH may lead to the identification of novel treatment targets for pain-related disability.

Prior studies have compared autonomic and central nervous system functioning between individuals with PTH and those with primary headache and pain-free controls [3,4,5,6,7,8,9,10,11]. When compared with migraineurs and pain-free controls, individuals with PTH have greater COMPASS-31 scores, which reflect elevated autonomic dysfunction across a variety of physiologic domains, including vasomotor function [12]. Additionally, these groups differ in the functional connectivity of prefrontal cortex (PFC) regions involved with pain processing [3]. Adaptations in the autonomic nervous system (ANS) may impact systemic responses to a noxious event [13], whereas altered function of the PFC may impact how a nociceptive experience is perceived or processed [4].

The cold pressor task (CPT) is a noxious experimental stimulus capable of challenging pain-related autonomic and cortical functions through cold-water immersion. The CPT causes a systemic hemodynamic response mediated by the influence of the ANS on cardiovascular function [14,15], and elicits a local hemodynamic response within the PFC associated with the cortical processing of pain [16,17]. Previously, the CPT has been associated with blunted and delayed autonomic responses in individuals with mild TBI [15] and deficient central pain modulation in those with pPTH [10].

Functional near-infrared spectroscopy (fNIRS) is a non-invasive neuroimaging tool that can be used to monitor both ANS [18] and cortical activity [19] using near-infrared light to assess changes in the blood oxygenation levels of tissues underlying optodes placed on the scalp. Shorter inter-optode distances preferentially measure hemodynamic changes in extracranial tissues that are regulated by the ANS [20]. Longer inter-optode distances additionally measure hemodynamic responses mediated by the intracranial activity of local cortical neurons [18]. Prior studies have shown that fNIRS can differentiate individuals with mmTBI [21,22] or migraine [23,24] from healthy controls during a variety of cognitive tasks. Although previously shown to detect hemodynamic responses to the CPT in pain-free individuals [25,26], fNIRS has not been used to investigate physiologic responses to noxious stimuli in indiviudals with pPTH.

This study sought to determine whether extracranial and intracranial hemodynamic responses to the CPT measured by short- and long-separation fNIRS channels over the PFC differ between individuals with pPTH and pain-free controls without a history of TBI or headache. Based on prior reports of autonomic dysregulation and impaired modulation of pain by the PFC in mmTBI and primary headache populations, we hypothesized that individuals with pPTH would exhibit blunted extracranial and intracranial hemodynamic responses to a noxious CPT compared to controls.

## 2. Materials and Methods

### 2.1. Participants

A subgroup of 10 veterans enrolled in a prior observational cohort study of pPTH [27] completed additional fNIRS testing for the present case–control investigation. Eligibility criteria and recruitment methods for participants with pPTH have been described previously [27]. Briefly, veterans with a history of pPTH following a mmTBI were recruited by convenience from the Veterans Administration San Diego Healthcare System (VASDHS). Screening was performed by telephone to confirm the presence of pPTH using diagnostic criteria from the International Classification of Headache Disorders-3 [28] which include both acute (within 7 days) and delayed onset (>7 days) of headaches following a traumatic head injury that persist for 3 or more months. Veterans 18 to 60 years of age who endorsed the onset of headaches within 12 months of a TBI diagnosis and were still experiencing headaches at the time of enrollment were eligible to participate. A post-injury onset interval of up to 12 months was based on prior literature showing that diagnostic criterion using a 7-day onset interval significantly underestimates rates of pPTH monitored prospectively in the first year after TBI [29].

Healthy controls (HC) were recruited by convenience through advertisements and registered lists of healthy research volunteers at the University of California, San Diego and the VASDHS. Eligible HC participants reported no lifetime history of concussion/TBI, persistent headache, or chronic pain. Ten HC (4 civilians, 3 veterans, and 3 active duty military) were matched to veterans with pPTH on age (±3 years), sex, and body mass index (BMI) (±3 kg/m^2^). Matching criteria were selected to account for the moderating effects of age and sex on the development and severity of pPTH [30,31] and to mitigate the impact of BMI on the accuracy of converting raw fNIRS signal intensities to relative changes in hemoglobin concentration [32,33].

pPTH candidates were excluded if they had a history of severe TBI or persistent head or neck pain prior to their first identified TBI. Exclusion criteria for both groups included (1) widespread interfering pain in multiple body regions other than the neck and head, (2) systemic (e.g., diabetes, lupus) or neurological (e.g., fibromyalgia, stroke) conditions potentially affecting sensation, (3) major psychiatric condition (e.g., bipolar disorder, psychosis), (4) current substance use disorder, (5) current use of opioids or other narcotics, or (6) pregnancy.

Eligible participants were scheduled for a single experimental session that included a clinical interview with surveys and quantitative sensory testing as described previously [27]. For the present study, participants underwent additional testing with fNIRS during performance of the CPT. All participants were asked to refrain from consuming substances known to impact pain processing, including non-prescription analgesic medication, cannabis, and alcohol, for 24 h before their research visit, which was confirmed by self-report. Testing was rescheduled for any participant who failed to adhere to these instructions or reported a headache episode on the day of testing. Written informed consent was obtained from all participants prior to enrollment in the study, which was conducted between August 2021 and January 2024. The study was conducted in accordance with procedures approved by the VASDHS Institutional Review Board and Research and Development Committee.

### 2.2. Procedures

All participants completed standardized surveys regarding their sociodemographics, medication use, TBI history, psychological characteristics, and headache symptoms. Social determinants, such as race/ethnicity, educational attainment, and marital status, were assessed due to known associations with altered pain processing states [34,35,36]. Routine use of prescription analgesic, psychotropic, and cardiovascular medication was documented. The Boston Assessment of TBI-Lifetime (BAT-L) was used to confirm TBI diagnosis and characterize injury characteristics [37,38]. The Neurobehavioral Symptom Inventory (NSI) (score range: 0–88 points) was used to determine the number and severity of current post-concussive symptoms, with higher scores indicating greater severity [39]. Symptoms of anxiety and depression were measured using the PROMIS Anxiety Short Form 8a and PROMIS Depression Short Form 8a, respectively [40]. The Pain Catastrophizing Scale (PCS) (score range: 0–52 points) was used to measure an individual’s belief that they will be negatively impacted by their pain [41]. A semi-structured headache history was conducted to determine the type and frequency of headaches, and the short-form Headache Impact Test (HIT-6) (score range: 36–78) was used to assess headache-related disability [42]. HIT-6 scores less than 49 indicate “little or no impact” of headache on daily life; 50–55 “some impact,” 56–59 “substantial impact,” and ≥60 “severe impact” [43]. The PROMIS Adult v2.0 Pain Intensity 3a survey assessed the intensity of headaches [44]. Higher scores for all outcome measures assessing clinical characteristics reflect greater levels of clinical severity. All PROMIS raw scores were converted to T-scores prior to comparing individuals with pPTH and HC. A PROMIS T-score of 50 is the average of the U.S. general population and every 10 units of change from 50 is one standard deviation [40].

**Figure 1:**
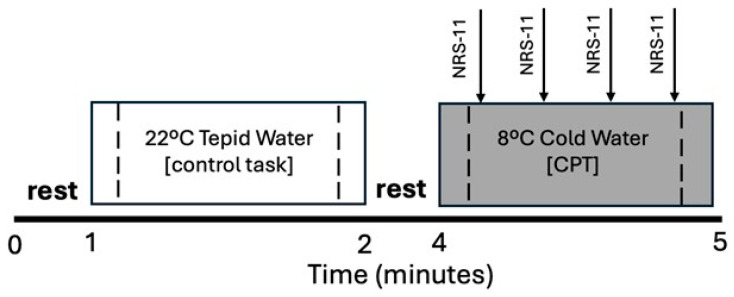
Experimental Protocol. Participants immersed their nondominant hand to wrist level in tepid water (22 °C for 1 min) followed by noxious cold water (8 °C for 1 min or until tolerance). Control and cold pressor tasks (CPT) were separated by a 2 min rest period. Verbal ratings of perceived pain intensity were collected every 15 s during the CPT using an 11-point numeric rating scale (NRS-11, arrows). Dashed lines indicate middle 75% of each task used to assess extra- and intracranial hemodynamic responses measured with functional near-infrared spectroscopy (fNIRS).

After completing clinical interviews and surveys, the Brite MKII fNIRS unit (Artinis Medical Systems B.V., Elst, The Netherlands) was used to monitor extra- and intracranial hemodynamic responses during control and experimental CPT procedures (Figure 1). The fNIRS device assessed oxygenated and deoxygenated hemoglobin using light emitted at 757 and 843 nm wavelengths, respectively. Data were collected at 50 Hz using Oxysoft software (Artinis Medical Systems B.V., The Netherlands). Transmitter and receiver optodes were placed 10 mm apart for the short separation channel (SSC) and 30 mm apart for each long separation channel (LSC). Channel positions were determined relative to the 10–20 system, and these positions were used to identify the approximate location of Brodmann’s areas (BA) and functional subdivisions of the PFC based on prior research [45]. Three LSCs were separately positioned over BA 8, 9/10, and 10 spanning the ipsilateral ventral and dorsal medial PFC (vmPFC and dmPFC, respectively), and 2 additional LSCs were positioned over the contralateral dmPFC at BA 8 and 9/10. These regions of interest were selected based on prior reports of their differential responsiveness to experimental pain in chronic pain populations [46,47,48,49,50,51,52,53,54,55] compared to controls [47,51,56,57,58,59,60,61,62,63]. Finally, a SSC positioned over the greater longitudinal fissure was used to monitor extracranial hemodynamic responses [20].

During the CPT, participants first submerged their nondominant hand to wrist level in 22 °C tepid water for one minute (control task) followed by submersion of the same hand to wrist level in an 8 °C circulating cold water bath for up to one minute or until tolerance (noxious cold pressor task). The control condition always preceded cold-water hand immersion because the CPT can have prolonged effects on physiologic responses [64]. Participants verbally rated their perceived pain intensity on a scale between 0 and 10 (11-point numeric rating scale, NRS-11) [65,66] every 15 s during cold-water hand immersion. A rating of “0” indicated “no pain”, whereas “10” indicated “the worst imaginable pain.” A pain rating of 10 was assigned to any time point that followed premature extraction of the hand (<60 s) due to cold pain intolerance. Evoked pain responses during the CPT were calculated as the change in NRS-11 ratings during the middle half of the CPT (NRS-11 rating at 45 s minus 15 s) to ensure that evoked pain responses were assessed within the measurement window used for fNIRS recordings. The evoked pain response during tepid water hand immersion was assigned an NRS-11 score of zero for the control condition.

### 2.3. Data Processing

**Figure 2:**
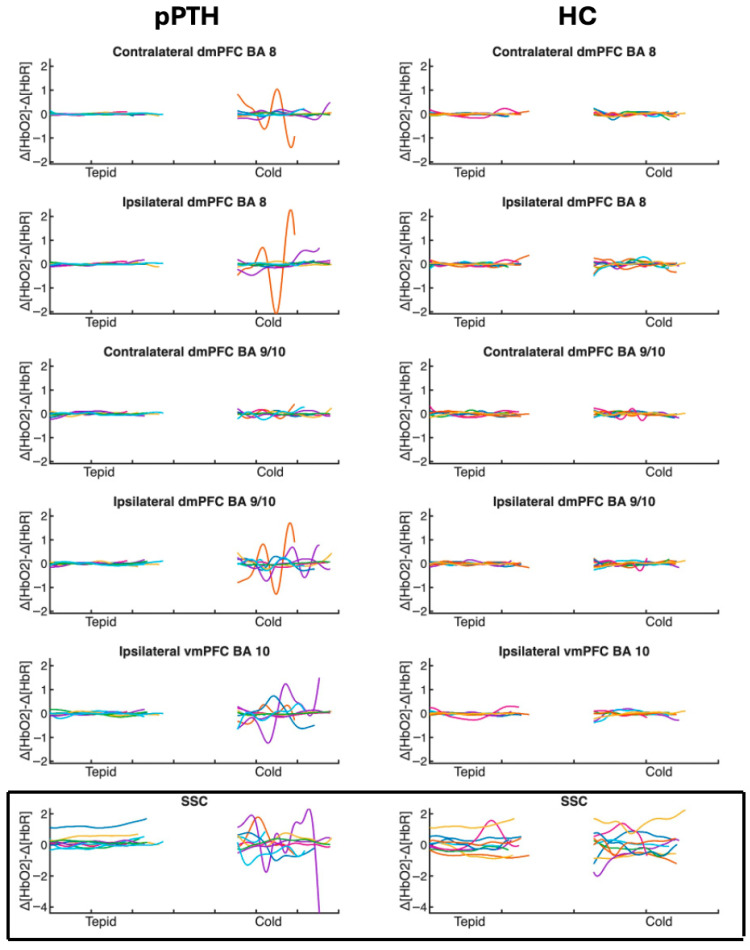
Time course of individual hemodynamic responses for participants with persistent post-traumatic headache and healthy controls during tepid- and cold-water hand immersion. Traces show the time course (*x*-axis) of individual hemodynamic responses (*y*-axis) measured as the difference between oxygenated [∆HbO_2_] and deoxygenated [∆HbO] hemoglobin concentrations at each time point. Each participant’s response is indicated with a different color. Difference values are plotted separately for each optode channel (rows) during the middle 75% of the control task (tepid-water hand immersion) and the experimental cold pressor task (cold-water hand immersion) for individual participants in the pPTH (**left column**) and HC (**right column**) groups. Peak-to-peak hemodynamic responses were calculated as the difference between the maxima and minima of each trace. Boxed and unboxed channels reflect extra- and intracranial hemodynamic responses, respectively. Abbreviations: BA = Brodmann’s Area; dmPFC = dorsomedial prefrontal cortex; vmPFC = ventromedial prefrontal cortex; SSC = short separation channel.

No signals were flagged by Oxysoft as having poor signal quality during data collection, and post hoc visual inspection of the raw traces revealed no motion artifact, clipping, or other evidence of non-physiologic noise during control and experimental tasks performed during quiet sitting. The modified-beer lambert law (MBLL) was implemented in Oxysoft to convert the differences in light intensity between pairs of transmitter and receiver optodes to relative changes in hemoglobin concentration [67]. A differential pathlength factor of 6 was used in the MBLL equation as recommended for adults [68]. A 0.01–0.09 Hz bandpass filter was applied to the raw signal [69] with no additional signal correction procedures. The SSC signal was then regressed out of each LSC signal to isolate intracranial responses in LSCs [70]. The relative change in deoxygenated hemoglobin (HbR) was then subtracted from the relative change in oxygenated hemoglobin (HbO2) concentration at each time point (∆[Hb02]−∆[HbR]) [71] to derive a difference signal for each channel. This difference is thought to reflect changes in the magnitude of cortical activation, given that [HbO2] and [HbR] measure oxygen supply and demand, respectively [71]. Finally, the peak-to-peak (PP) magnitude of the evoked hemodynamic response ((∆[Hb02]−∆[HbR])_PP_) was calculated as the difference between the maxima and minima of the derived difference signal, as illustrated in Figure 2.

Signal processing was conducted using custom software developed in MATLAB Online (V2023a, MathWorks, Natick, MA, USA). Responses that exceeded three standard deviations from the mean were identified as outliers for sensitivity analyses (ipsilateral dmPFC BA8 (N = 1); contralateral dmPFC BA9/10 (N = 2); ipsilateral vmPFC BA 10 (N = 2); SSC (N = 1)). Hemodynamic responses were analyzed separately for tepid- and cold-water conditions, using data from the middle 75% of each task to minimize anticipatory effects and movement artifacts associated with task transitions.

### 2.4. Data Analysis

Descriptive statistics were calculated for TBI, sociodemographic, and clinical characteristics and for evoked pain responses during the CPT. Group differences were then assessed using independent t-tests and chi-square tests for continuous and categorical data, respectively. A Mann–Whitney U test was performed if the assumption of normality as determined by the Shapiro–Wilk test was not met. To assess representativeness of the clinical sample, similar procedures were used to compare TBI and headache characteristics for the subgroup of participants with pPTH enrolled in the present study with those of the larger pPTH cohort in our prior investigation [27].

Separate linear mixed effects models were used to analyze group differences in the PP hemodynamic response during the tepid- and cold-water tasks for each channel. The Shapiro–Wilk test indicated that the assumption of normality was violated for model residuals; therefore, a log transformation was applied to the PP hemodynamic responses for each channel. Participants were treated as a random effect to account for individual differences in blood supply to the scalp and brain. Fixed effects included task (tepid, cold), group (pPTH, HC), evoked pain (NRS-11), and the interaction between task and group. Task was treated as a repeated measures variable with the log of the PP hemodynamic response during tepid- and cold-water hand immersion as separate time points. A Benjamini–Hochberg correction was performed to control for multiple comparisons, and sensitivity analyses were performed with and without outliers included.

No a priori power analysis was conducted for this pilot investigation which was limited to recruitment of a subsample of participants enrolled in the parent study. To inform future investigations, effect sizes were calculated between tepid- and cold-water hand immersion for each group separately to quantify the magnitude of the CPT effect on hemodynamic responses. Cohen’s d < 0.20 was interpreted as a negligible effect, 0.20–0.49 small, 0.50–0.79 medium, and ≥0.80 large [72]. Additionally, a post hoc power analysis (α = 0.05, power = 0.80) was performed using computed effect sizes for between-group differences in the CPT effect to determine the number of participants that would be required to reliably identify group differences for each LSC and SSC. All data were analyzed using RStudio (Version 2025.09.2+418; RStudio Team, Boston, MA, USA, 2023).

## 3. Results

### 3.1. Traumatic Brain Injury and Headache Characteristics

Table 1 shows TBI and headache characteristics for participants with pPTH. All participants with pPTH experienced more than one mmTBI during their lifetime (range 2 to 20), with the most severe mmTBI occurring 2 to 27 years prior to study enrollment. The majority of participants experienced a TBI of mild severity associated with both a loss of consciousness and altered mental status. The most common mechanism of injury was a motor vehicle accident. Most participants experienced a delayed onset of headaches after injury and reported mixed symptoms characteristic of both migraine and tension-type headache. All individuals with pPTH reported having a headache at least once per week and 80% routinely experienced headaches several times per week. HIT-6 and PROMIS scores indicated that pPTH had a substantial impact on daily life, with headache intensity exceeding that of general population. Forty percent of participants reported using prescription analgesic medications, 60% used psychotropic medications, and 20% used cardiovascular medications. These TBI and headache characteristics were similar to those observed for the larger clinical cohort of veterans with pPTH described in our previous study (*p* ≥ 0.38) [27].

### 3.2. Sociodemographic and Psychologic Characteristics

As shown in Table 2, sociodemographic characteristics were not significantly different between the pPTH and HC groups. Matched groups were 90% male and differed in age by 0.2 years and BMI by 0.18 kg/m^2^ on average. Across groups, 60% of the sample identified as non-Hispanic white, 50% had a bachelor’s degree or higher, and 70% were married or partnered. Compared to the HC group, the pPTH group reported significantly greater levels of anxiety, depression, and pain catastrophizing (*p* ≤ 0.001). On average, severity scores for the pPTH group indicated clinically significant symptoms of anxiety and depression, with low levels of pain catastrophizing.

### 3.3. Peak-to-Peak Hemodynamic Responses

**Figure 3:**
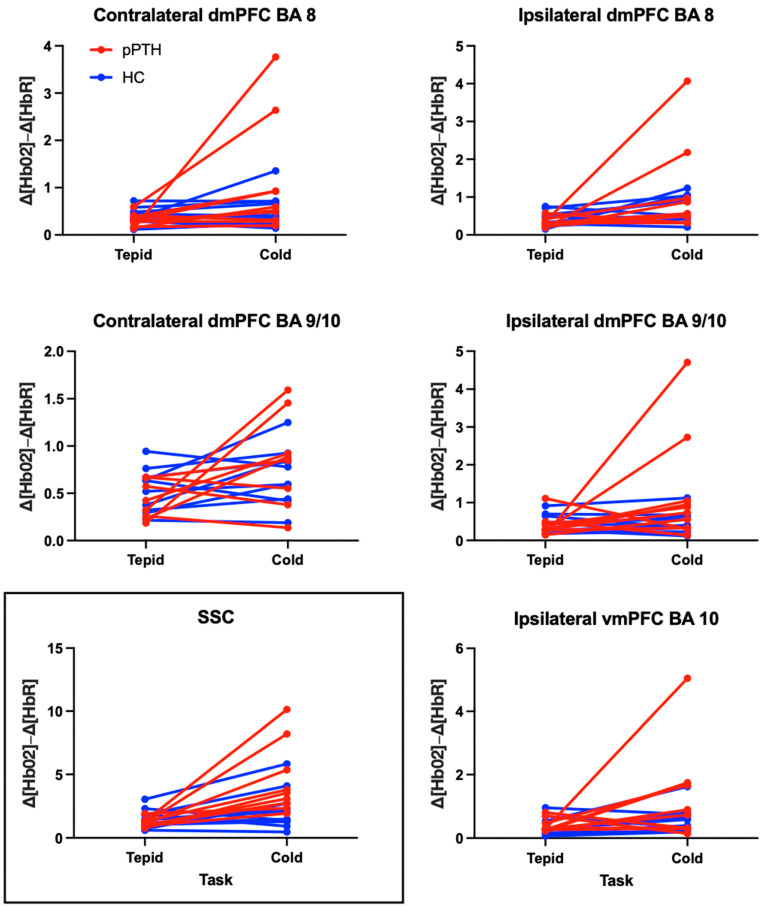
Differences in peak-to-peak hemodynamic responses for participants with persistent post-traumatic headache and healthy controls during tepid- and cold-water hand immersion. Each line reflects a single participant in the persistent post-traumatic headache (pPTH; red) or healthy control (HC; blue) groups. Plots show changes in peak-to-peak hemodynamic responses between tepid- (**left column**) and cold-water hand immersion (**right column**) prior to logarithmic adjustment for non-normality. Boxed and unboxed channels reflect extra- and intracranial hemodynamic responses, respectively. Abbreviations: BA = Brodmann’s Area; dmPFC = dorsomedial prefrontal cortex; vmPFC = ventromedial prefrontal cortex; SSC = short separation channel.

Table 3 provides results from the linear mixed-effects model for each fNIRS channel, with outliers excluded. These results remained unchanged when outliers were included in the sensitivity analysis. The main effects of group and task were non-significant for all channels, whereas the interaction effect of group by task was significant only for the SSC (*p* = 0.031). A significant interaction for the SSC indicated that the pPTH group had a greater extracranial hemodynamic response during cold-water hand immersion compared to tepid-water hand immersion, whereas the HC group showed a negligible difference in extracranial hemodynamic responses between tasks (Figure 3).

Consistent with a significant interaction effect for the SSC, the within group effect size for the extracranial hemodynamic response to cold-water hand immersion was large for the pPTH group (d = 2.86 (1.49, 4.19)) in contrast to a small and non-significant effect size for the HC group (d = 0.41 (−0.48, 1.29)). Both groups showed large and significant effects of the CPT on intracranial hemodynamic responses for the ipsilateral dmPFC BA 8 (pPTH: d = 1.42 (0.35, 2.44), HC: d = 0.99 (0.05, 1.92)). Within group effect sizes for other LSCs were large and significant only for the pPTH group: contralateral dmPFC BA 8 (pPTH: d = 1.23 (0.25, 2.18), HC: d = 0.25 (−0.63, 1.13)); ipsilateral dmPFC BA 9/10 (pPTH: d = 1.07 (0.12, 2.00), HC: d= 0.05 (−0.83, 0.92)); ipsilateral vmPFC BA 10 (pPTH: d = 0.94 (0.00, 1.86), HC: d = 0.83 (−0.15, 0.77)). CPT effects on hemodynamic responses in the contralateral dmPFC BA 9/10 were large for the pPTH group and medium for the HC group, but these effects were non-significant for both groups (pPTH: d = 0.89 (−0.16, 1.91), HC: d = 0.46 (−0.44, 1.34)). The sample sizes required to detect group differences with 80% power include: SSC = 4; ipsilateral dmPFC BA 9/10 = 17; contralateral dmPFC BA 8 = 18; ipsilateral dmPFC BA 8 = 86; contralateral dmPFC BA 9/10 = 86; and ipsilateral vmPFC BA 10 = 1299.

### 3.4. Evoked Pain Responses

Evoked pain responses during cold-water hand immersion were not different (W = 42, *p* = 0.600) between pPTH (s = 3.6, SE = 0.7) and HC (s = 3.0, SE = 0.4) groups. Two channels had significant coefficients for the evoked pain response, including the ipsilateral dmPFC (BA 8; *p* = 0.031) and ipsilateral dmPFC BA 9/10 (*p* = 0.031). All significant coefficients ranged between 0.22 and 0.27; therefore, a greater increase in pain intensity during cold-water hand immersion was associated with a greater hemodynamic response for the ipsilateral dmPFC regardless of group membership.

## 4. Discussion

This study investigated whether intra- and extracranial hemodynamic responses differed between individuals with pPTH and HCs during a noxious CPT. Whereas both groups had similar levels of evoked pain and intracranial PFC responses to the CPT, extracranial hemodynamic responses were greater in individuals with pPTH. These findings suggest that individuals with pPTH may exhibit heightened systemic autonomic responses to noxious cold stimuli compared to healthy individuals. Further, we found larger evoked pain responses during the CPT to be associated with greater hemodynamic responses within the dmPFC regardless of group membership. This suggests that pain evoked by the CPT is related to changes in activity of the medial PFC rather than the ANS in individuals with and without pPTH.

### 4.1. Hemodynamic Responses to the Cold Pressor Task Measured by fNIRS

Prior studies using fNIRS to investigate hemodynamic responses to the CPT have focused primarily on nociceptive [25,26,73] and cognitive [74,75,76,77] functioning in neurologically intact, pain-free adults. Despite using a variety of metrics to quantify hemodynamic responses during the CPT, these studies have consistently demonstrated robust activation of both short- and long-separation fNIRS channels over the forehead. Within-group hemodynamic responses to the CPT observed for the dmPFC in the present study are consistent with prior studies of healthy adults [25,26,73,74,75,76,77]; however, the size of the effect was notably smaller for HCs in our study compared to a similar study by Barati et al. (2013) [25]. This difference may be due to our use of a more conservative frequency range for filtering the fNIRS signal, an alternative measure of the hemodynamic response (∆[Total Hb] vs. (∆[Hb02]−∆[HbR])), or our removal of extra-cranial hemodynamic activity from LSC signals prior to evaluating the magnitude of intra-cranial responses [70]. It is notable that LSCs and SSCs were evaluated separately by Barati et al. (2013) [25], which may not account for the confounding effects of extra-cranial signal changes. Together, these findings support prior research suggesting that systemic hemodynamic responses may account for a substantial proportion of the LSC signal measured during tasks such as the CPT which activate a robust autonomic response. [14]

To our knowledge, only two studies have investigated hemodynamic responses to the CPT in clinical populations. One study reported an increase in combined extra- and intracranial oxygenated hemoglobin (i.e., systemic and/or cortical hyperactivation) during the CPT in individuals with fibromyalgia compared to healthy controls [78]. Another study reported reduced intracranial oxygenated hemoglobin (i.e., cortical hypoactivation) of the ipsilateral dlPFC during cold-water hand immersion performed under social evaluative threat in individuals with major depressive disorder (MDD) compared to healthy controls [79]. The present investigation is the first to report elevated extracranial hemodynamic responses to the CPT with no difference in responses of the medial PFC in individuals with pPTH compared to HCs. Our findings for pPTH are consistent with those for fibromyalgia but contrast with those for MDD, suggesting that elevated hemodynamic responses to noxious stimuli may be a more general feature of chronic pain conditions but not depressive symptoms which are common among those with chronic pain. Consistent with prior studies on pPTH and chronic headache [3,30,80], our pPTH sample reported more severe symptoms of anxiety, depression, and pain catastrophizing compared to controls. While these psychological characteristics have been associated with impaired nociceptive processing [81,82,83] and altered hemodynamic activity at BA 9 and 10 assessed with MRI and PET in prior studies [84,85,86,87,88], they did not result in significant group differences in either evoked pain or intracranial hemodynamic responses to the CPT in our sample. Additional research is needed to better understand clinical correlates of altered hemodynamic responses in conditions with overlapping symptomatology.

### 4.2. Role of ANS Regulation on Extracranial Hemodynamic Responses

The ANS is important to the maintenance of homeostasis in the human body. It comprises the sympathetic and parasympathetic nervous systems, which are responsible for mobilizing energy in times of increased or reduced levels of stress, respectively [89]. During nociception, the ANS helps support the metabolic requirements of behaviors that address threatening stimuli by increasing sympathetic tone. These sympathetically mediated responses to nociception are found during the CPT and include elevated levels of cardiac output and blood pressure [14]. Because cardiovascular responses during the CPT are associated with skin blood flow changes at the forehead [64], our measurement of extracranial hemodynamic responses via the SSC may reflect changes in the ANS response to a noxious event. Prior studies of healthy individuals found that the CPT induces an increase in extracranial blood flow, and these changes are associated with an increase in sympathetic tone [64,90]. Other research suggests that extracranial hemodynamic responses are likely mediated by sympathetic neurons within cutaneous tissue [90]. Collectively, this evidence may suggest that compared to HCs, individuals with pPTH have an exaggerated sympathetic response to the CPT. Previous CPT studies have found migraine to be associated with greater cardiovascular responses [91] and elevated levels of skin blood flow at the forehead compared to controls [92], whereas individuals with post-concussion symptoms after a mild TBI have shown attenuated cardiovascular responses to the CPT [15]. Thus, heightened extracranial hemodynamic responses observed in the present study may be more related to the presence of headache than history of head trauma.

Impaired ANS function has been linked to increased clinical severity of migraine [93]. Cognitive behavioral therapies (CBT) have been shown to improve ANS function [94,95,96] and reduce headache-related disability in individuals with pPTH [97], suggesting that CBT may improve the clinical severity of headache in individuals with pPTH by enhancing ANS regulation. fNIRS may provide a useful biomarker to examine changes in ANS function following CBT and other therapies that target autonomic regulation in the management of pPTH.

### 4.3. Relationship Between Evoked Pain and Intracranial Hemodynamic Responses of the Medial PFC

During nociception, the PFC integrates information from cognitive, affective–motivational, and sensoridiscriminative domains to produce and modulate pain-related behavior [98]. Metrics derived from the hemodynamic signal recorded over the PFC with fNIRS have previously been found to correlate with the threshold and intensity of pain during the CPT in healthy individuals [25,26]. Our study found no difference in intracranial hemodynamic responses to the CPT for LSCs at the vmPFC or dmPFC in individuals with pPTH compared to HCs. We also found no group difference in the intensity of pain evoked by the CPT, suggesting that processing of noxious pain stimuli by the medial PFC may be similar for individuals with and without pPTH.

Higher levels of evoked pain during the CPT were related to greater intracranial hemodynamic responses for the ipsilateral dmPFC regardless of group membership. Notably, evoked pain was not associated with extracranial hemodynamic responses measured by the SSC, suggesting that pain evoked by the CPT is related to medial PFC rather than ANS function. This finding is in contrast to Barati et al. [25,26] who reported significant associations between pain evoked by the CPT and hemodynamic responses in both short- and long-separation channels in healthy adults. Additional studies with larger samples are needed to clarify whether these associations may be population dependent. 

### 4.4. Study Limitations and Future Research Directions

Due to the small sample of individuals with pPTH who agreed to participate, our study may have been underpowered to detect more subtle group effects on intracranial hemodynamic responses to the CPT, specifically within dmPFC BA 8 and 9/10. Future studies with larger samples are needed to investigate possible impairments in nociceptive processing by the dmPFC among individuals with pPTH. The small sample size also limited our analyses to group comparisons without further consideration of potential covariates. While this limitation was partially mitigated by matching participants on confounding characteristics such as age and sex, future research should enroll a larger sample of participants to determine whether hemodynamic responses to the CPT are related to clinical, psychological, or other characteristics that differ between individuals with pPTH and HCs. This is particularly important given that our control group included active duty military and civilians who likely differ from veterans with regard to occupational and environmental stress exposures [99].

Given prior evidence of autonomic dysregulation in individuals with post-concussion syndrome after mild TBI [100] and in those with chronic headache [101], future studies should include comparator groups with non-traumatic headache and headache-free individuals with mild TBI to better understand whether elevated extracranial hemodynamic responses to a noxious cold stimulus are a consequence of head trauma, headache, or their interaction.

Changes in extracranial blood flow are thought to reflect modulation by the ANS, but local physiology can additionally impact blood flow [102,103] and may have contributed to group differences in extracranial hemodynamic responses during the CPT. For example, elevated extracranial blood flow in individuals with pPTH compared to HCs may be related to augmented local metabolism or impaired arteriole autoregulation within the scalp. To more directly examine the role of ANS dysfunction, future research should assess the relationship between extracranial hemodynamic responses and systemic markers of ANS function during the CPT, including changes in heart rate, mean arterial pressure, heart rate variability, electrodermal activity, and respiratory rate [104,105,106,107,108,109].

Evoked pain intensity was the only pain behavior assessed during the CPT; therefore, interpretations were restricted to this pain processing construct. Future research should additionally examine pain unpleasantness and conditioned pain modulation, which are also related to the function of BAs 9 and 10 [98,110,111,112] and reflect the affective–motivational and pain modulatory dimensions of pain processing, respectively. Better understanding which dimensions of pain processing are related to hemodynamic responses during the CPT may enhance our understanding of mechanisms underlying cortical–affective coupling to help inform treatment targets for pPTH.

BAs and functional subdivisions were assigned to channels based on their relationship to landmarks within the 10–20 system. Future studies should use tools, such as magnetic resonance imaging [113] or neuronavigation devices, to advance the methodologic accuracy of spatially registering anatomical structures at the individual level. Future research could also investigate differences in activation of other regions within the PFC, such as the dorsolateral PFC [114], or differences in functional connectivity between the PFC and pain processing network nodes previously implicated in pPTH [3,115,116,117,118].

## 5. Conclusions

Individuals with pPTH have greater extracranial hemodynamic responses to a noxious CPT than HCs. These differences may reflect heightened systemic autonomic responses to noxious stimuli in individuals with pPTH and should be explored in future studies as a biomarker for subgroup identification and longitudinal responses to treatment. Further, hemodynamic responses measured with fNIRS over the dmPFC during cold-water hand immersion are associated with perceived pain intensity and may provide a useful biomarker for future investigations of individual variations in pain processing for both healthy and clinical populations.

## Figures and Tables

**Table 1:** Traumatic brain injury and headache characteristics for participants with post-traumatic headache.

**NSI** (0-88 points)	36.5 (15.2)
**BAT-L** (0–60 points)	5.9 (2.6)
**Number of lifetime TBIs ^a^**	3.5 (4.8)
**Time since TBI ^b^** (months)	143.8 (92.7)
**TBI severity grade ^b^**, n (%)	
Mild	
Grade I	1 (10)
Grade II	6 (60)
Grade III	2 (20)
Moderate	1 (10)
**LOC ^b^**, n (%)	7 (70)
**AMS ^b^**, n (%)	9 (90)
**Mechanism of injury ^b^**, n (%)	
Blast	2 (20)
Blunt	8 (80)
**TBI sustained during Combat/Deployment TBI**, n (%)	8 (80)
**Headache Characteristics**	
**Onset of headache after TBI ^b^**, n (%)	
Acute (≤7 days)	3 (30)
Delayed (>7 days)	7 (70)
**HIT-6** (36–78 points)	57.3 (10.0)
**PROMIS headache intensity** (T-score)	58.2 (7.6)

Values are mean (SD) or n (%) unless otherwise indicated as ^a^ median (IQR) ^b^ Reported for most severe TBI only. Abbreviations: AMS = altered mental status, BAT-L = Boston Assessment of Traumatic Brain Injury-Lifetime, HIT-6 = Headache Impact Test, LOC = loss of consciousness, NSI = Neurobehavioral Symptom Inventory, PROMIS = Patient-Reported Outcomes Measurement Information System, TBI = traumatic brain injury.

**Table 2:** Comparison of sociodemographic and psychological characteristics between groups with persistent post-traumatic headache and healthy controls.

	pPTH Group	HC Group	*p*-Value
**Sociodemographic Characteristics**			
**Sex**, n (%)			1.000
Male	9 (90)	9 (90)	
Female	1 (10)	1 (10)	
**Age**			0.968
Mean (+/− SD), years	39.8 (11.0)	39.6 (11.0)	
**BMI**			0.520
Mean (+/− SD), kg/m^2^	28.8 (3.2)	29.0 (2.3)	
**Ethnicity/Race**, n (%)			0.867
Non-Hispanic White	5 (50)	7 (70)	
Non-Hispanic Black	2 (20)	1 (10)	
Hispanic White	3 (30)	2 (20)	
**Education**, n (%)			0.339
High school diploma or less	1 (10)	2 (20)	
Some college	5 (50)	2 (20)	
Bachelor’s degree or higher	4 (40)	6 (60)	
**Marital status**, n (%)			0.223
Married or partnered	7 (70)	7 (70)	
Single	1 (10)	3 (30)	
Divorced	2 (20)	0 (0)	
**Psychological Characteristics**			
**PROMIS Anxiety** (T-score)	61.1 (4.9)	47.0 (7.4)	0.001
**PROMIS Depression** (T-score)	56.0 (6.1)	41.3 (4.1)	<0.001
**PCS** (0–52 points)	16.3 (10.1)	1.6 (3.4)	<0.001

Abbreviations: BMI = body mass index, HC = healthy control, PCS= Pain Catastrophizing Scale, pPTH = persistent post-traumatic headache, PROMIS = Patient-Reported Outcomes Measurement Information System.

**Table 3:** Mixed-effects model for peak-to-peak hemodynamic responses to a noxious cold pressor task.

	Cont. dmPFC BA 8			lps. dmPFC BA 8			Cont. dmPFC BA 9/10			lps. dmPFC BA 9/10			lps. vmPFC BA 10			SSC		
**Fixed Effects**	**β**	**CI**	***p***	**β**	**CI**	***p***	**β**	**CI**	***p***	**β**	**CI**	***p***	**β**	**CI**	***p***	**β**	**CI**	***p***
Evoked Pain	0.16	(0.01, 0.31)	0.184	0.22	(0.09, 0.35)	**0.031**	0.19	(0.06, 0.33)	0.069	0.27	(0.11, 0.42)	**0.** **031**	0.21	(0.01, 0.41)	0.184	0.12	(−0.01, 0.25)	0.224
Task	−0.34	(−1.02, 0.33)	0.554	−0.13	(−0.73, 0.47)	0.78	−0.35	(−0.95, 0.24)	0.554	−0.78	(−1.49, −0.06)	0.184	0.06	(−0.80, 0.92)	0.936	−0.10	(−0.64, 0.42)	0.797
Group	−0.29	(−0.84, 0.25)	0.554	−0.21	(−0.66, 0.25)	0.554	−0.26	(−0.73, 0.21)	0.554	−0.24	(−0.79, 0.30)	0.554	0.24	(−0.42, 0.90)	0.597	−0.20	(−0.65, 0.25)	0.554
Task × Group	0.67	(−0.05, 1.38)	0.224	0.33	(−0.32, 0.98)	0.554	0.27	(−0.39, 0.91)	0.563	0.71	(−0.06, 1.49)	0.224	−0.03	(−0.98, 0.91)	0.946	0.95	(0.41, 1.48)	**0.031**

Significant findings are indicated in bold font (*p* < 0.05). Task: Tepid-water hand immersion (reference) versus cold-water hand immersion; Group: Healthy control (reference) versus persistent post-traumatic headache. Abbreviations: BA = Brodmann’s Area, Cont. = contralateral, dmPFC = dorsomedial prefrontal cortex, Ips. = ipsilateral, SSC = short separation channel, vmPFC = ventromedial prefrontal cortex.

## Data Availability

The data presented in this study are available on request from the corresponding author due to data sharing policies at the VA.

## References

[1] Kothari S.F., Eggertsen P.P., Frederiksen O.V., Thastum M.M., Svendsen S.W., Tuborgh A., Næss-Schmidt E.T., Rask C.U., Schröder A., Kasch H. (2022). Characterization of persistent post-traumatic headache and management strategies in adolescents and young adults following mild traumatic brain injury. Sci. Rep..

[2] Herrero Babiloni A., Bouferguene Y., Exposto F.G., Beauregard R., Lavigne G.J., Moana-Filho E.J., Arbour C. (2023). The prevalence of persistent post-traumatic headache in adult civilian traumatic brain injury: A systematic review and meta-analysis on the past 14 years. Pain.

[3] Dumkrieger G., Chong C.D., Ross K., Berisha V., Schwedt T.J. (2019). Static and dynamic functional connectivity differences between migraine and persistent post-traumatic headache: A resting-state magnetic resonance imaging study. Cephalalgia.

[4] Chong C.D., Peplinski J., Berisha V., Ross K., Schwedt T.J. (2019). Differences in fibertract profiles between patients with migraine and those with persistent post-traumatic headache. Cephalalgia.

[5] Ashina H., Al-Khazali H.M., Iljazi A., Ashina S., Amin F.M., Schytz H.W. (2022). Total tenderness score and pressure pain thresholds in persistent post-traumatic headache attributed to mild traumatic brain injury. J. Headache Pain.

[6] Levy D., Gruener H., Riabinin M., Feingold Y., Schreiber S., Pick C.G., Defrin R. (2020). Different clinical phenotypes of persistent post-traumatic headache exhibit distinct sensory profiles. Cephalalgia.

[7] Niu X., Bai L., Sun Y., Wang S., Cao J., Sun C., Wang Z., Xu H., Gan S., Fan G. (2019). Disruption of periaqueductal grey-default mode network functional connectivity predicts persistent post-traumatic headache in mild traumatic brain injury. J Neurol. Neurosurg. Psychiatry.

[8] Defrin R., Riabinin M., Feingold Y., Schreiber S., Pick C.G. (2015). Deficient pain modulatory systems in patients with mild traumatic brain and chronic post-traumatic headache: Implications for its mechanism. J. Neurotrauma.

[9] Defrin R., Gruener H., Schreiber S., Pick C.G. (2010). Quantitative somatosensory testing of subjects with chronic post-traumatic headache: Implications on its mechanisms. Eur. J. Pain.

[10] Naugle K.M., Carey C., Evans E., Saxe J., Overman R., White F.A. (2020). The role of deficient pain modulatory systems in the development of persistent post-traumatic headaches following mild traumatic brain injury: An exploratory longitudinal study. J. Headache Pain.

[11] Starling A.J., Cortez M.M., Jarvis N.R., Zhang N., Porreca F., Chong C.D., Schwedt T.J. (2022). Cutaneous heat and light-induced pain thresholds in post-traumatic headache attributed to mild traumatic brain injury. Headache.

[12] Howard L., Dumkrieger G., Chong C.D., Ross K., Berisha V., Schwedt T.J. (2018). Symptoms of Autonomic Dysfunction Among Those With Persistent Posttraumatic Headache Attributed to Mild Traumatic Brain Injury: A Comparison to Migraine and Healthy Controls. Headache.

[13] Burton A.R., Fazalbhoy A., Macefield V.G. (2016). Sympathetic Responses to Noxious Stimulation of Muscle and Skin. Front. Neurol..

[14] Lamotte G., Boes C.J., Low P.A., Coon E.A. (2021). The expanding role of the cold pressor test: A brief history. Clin. Auton. Res..

[15] Johnson B.D., Sackett J.R., Schlader Z.J., Leddy J.J. (2020). Attenuated Cardiovascular Responses to the Cold Pressor Test in Concussed Collegiate Athletes. J. Athl. Train.

[16] La Cesa S., Tinelli E., Toschi N., Di Stefano G., Collorone S., Aceti A., Francia A., Cruccu G., Truini A., Caramia F. (2014). fMRI pain activation in the periaqueductal gray in healthy volunteers during the cold pressor test. Magn. Reson. Imaging.

[17] Lapotka M., Ruz M., Salamanca Ballesteros A., Ocon Hernandez O. (2017). Cold pressor gel test: A safe alternative to the cold pressor test in fMRI. Magn. Reson. Med..

[18] Chen W.L., Wagner J., Heugel N., Sugar J., Lee Y.W., Conant L., Malloy M., Heffernan J., Quirk B., Zinos A. (2020). Functional Near-Infrared Spectroscopy and Its Clinical Application in the Field of Neuroscience: Advances and Future Directions. Front. Neurosci..

[19] Masataka N., Perlovsky L., Hiraki K. (2015). Near-infrared spectroscopy (NIRS) in functional research of prefrontal cortex. Front. Hum. Neurosci..

[20] Zhou X., Sobczak G., McKay C.M., Litovsky R.Y. (2020). Comparing fNIRS signal qualities between approaches with and without short channels. PLoS ONE.

[21] Chang F., Li H., Li N., Zhang S., Liu C., Zhang Q., Cai W. (2022). Functional near-infrared spectroscopy as a potential objective evaluation technique in neurocognitive disorders after traumatic brain injury. Front. Psychiatry.

[22] Skau S., Bunketorp-Kall L., Kuhn H.G., Johansson B. (2019). Mental Fatigue and Functional Near-Infrared Spectroscopy (fNIRS)—Based Assessment of Cognitive Performance After Mild Traumatic Brain Injury. Front. Hum. Neurosci..

[23] Chen W.T., Hsieh C.Y., Liu Y.H., Cheong P.L., Wang Y.M., Sun C.W. (2022). Migraine classification by machine learning with functional near-infrared spectroscopy during the mental arithmetic task. Sci. Rep..

[24] Chen W.-T., Li C.-C., Liu Y.-H., Cheong P.-L., Wang Y.-M., Sun C.-W. (2025). Migraine Detection in Young Group Based on Functional Near-Infrared Spectroscopy Measurements. IEEE J. Sel. Top. Quantum Electron..

[25] Barati Z., Shewokis P.A., Izzetoglu M., Polikar R., Mychaskiw G., Pourrezaei K. (2013). Hemodynamic response to repeated noxious cold pressor tests measured by functional near infrared spectroscopy on forehead. Ann. Biomed. Eng..

[26] Barati Z., Zakeri I., Pourrezaei K. (2017). Functional near-infrared spectroscopy study on tonic pain activation by cold pressor test. Neurophotonics.

[27] Monroe K., Schiehser D., Parr A., Simmons A., Hays W., Bailey B., Shahidi B. (2025). Biological markers of brain network connectivity and pain sensitivity distinguish low coping from high coping Veterans with persistent post-traumatic headache. J. Neurotrauma.

[28] (2018). Headache Classification Committee of the International Headache Society (IHS) The International Classification of Headache Disorders, 3rd edition. Cephalalgia.

[29] Hoffman J.M., Lucas S., Dikmen S., Braden C.A., Brown A.W., Brunner R., Diaz-Arrastia R., Walker W.C., Watanabe T.K., Bell K.R. (2011). Natural history of headache after traumatic brain injury. J. Neurotrauma.

[30] Yilmaz T., Roks G., de Koning M., Scheenen M., van der Horn H., Plas G., Hageman G., Schoonman G., Spikman J., van der Naalt J. (2017). Risk factors and outcomes associated with post-traumatic headache after mild traumatic brain injury. Emerg. Med. J..

[31] Sawyer K., Bell K.R., Ehde D.M., Temkin N., Dikmen S., Williams R.M., Dillworth T., Hoffman J.M. (2015). Longitudinal Study of Headache Trajectories in the Year After Mild Traumatic Brain Injury: Relation to Posttraumatic Stress Disorder Symptoms. Arch. Phys. Med. Rehabil..

[32] Gorniak S.L., Meng H., Pollonini L. (2022). Correlation between subcutaneous adipose tissue of the head and body mass index: Implications for functional neuroimaging. Hum. Mov. Sci..

[33] Haeussinger F.B., Heinzel S., Hahn T., Schecklmann M., Ehlis A.C., Fallgatter A.J. (2011). Simulation of near-infrared light absorption considering individual head and prefrontal cortex anatomy: Implications for optical neuroimaging. PLoS ONE.

[34] Maracci L.M., Rodrigues A.S., Knorst J.K., Salbego R.S., Ferrazzo V.A., Liedke G.S., Silva T.B., Marquezan M. (2022). Does marital status influence TMD-related chronic pain? A cross-sectional study. J. Bodyw. Mov. Ther..

[35] Topping M., Fletcher J. (2024). Educational attainment, family background and the emergence of pain gradients in adulthood. Soc. Sci. Med..

[36] Rahim-Williams B., Riley J.L., Williams A.K., Fillingim R.B. (2012). A quantitative review of ethnic group differences in experimental pain response: Do biology, psychology, and culture matter?. Pain Med..

[37] Fortier C.B., Amick M.M., Grande L., McGlynn S., Kenna A., Morra L., Clark A., Milberg W.P., McGlinchey R.E. (2014). The Boston Assessment of Traumatic Brain Injury-Lifetime (BAT-L) semistructured interview: Evidence of research utility and validity. J. Head Trauma Rehabil..

[38] Kim S., Currao A., Fonda J.R., Beck B., Kenna A., Fortier C.B. (2023). Diagnostic Accuracy of the Boston Assessment of Traumatic Brain Injury-Lifetime Clinical Interview Compared to Department of Defense Medical Records. Mil. Med..

[39] Soble J.R., Silva M.A., Vanderploeg R.D., Curtiss G., Belanger H.G., Donnell A.J., Scott S.G. (2014). Normative Data for the Neurobehavioral Symptom Inventory (NSI) and post-concussion symptom profiles among TBI, PTSD, and nonclinical samples. Clin. Neuropsychol..

[40] Pilkonis P.A., Choi S.W., Reise S.P., Stover A.M., Riley W.T., Cella D., Group P.C. (2011). Item banks for measuring emotional distress from the Patient-Reported Outcomes Measurement Information System (PROMIS(R)): Depression, anxiety, and anger. Assessment.

[41] Osman A., Barrios F., Kopper B., Hauptmann W., Jones J., O’’Neill E. (1997). Factor Structure, Reliability, and Validity of the Pain Catastrophizing Scale. J. Behav. Med..

[42] Kosinski M., Bayliss M.S., Bjorner J.B., Ware J.E., Garber W.H., Batenhorst A., Cady R., Dahlöf C.G.H., Dowson A., Tepper S. (2003). A six-item short-form survey for measuring headache impact: The HIT-6. Qual. Life Res..

[43] Yang M., Rendas-Baum R., Varon S.F., Kosinski M. (2011). Validation of the Headache Impact Test (HIT-6) across episodic and chronic migraine. Cephalalgia.

[44] HealthMeasures (2020). Pain Intensity A Brief Guide to the PROMIS Pain Intensity Instrunments.

[45] Scrivener C.L., Reader A.T. (2022). Variability of EEG electrode positions and their underlying brain regions: Visualizing gel artifacts from a simultaneous EEG-fMRI dataset. Brain Behav..

[46] Cook D., Lange G., Ciccone D., Liu W., Steffener J., Natelson B. (2004). Functional Imaging of Pain in Patients with Primary Fibromyalgia. J. Rheumatol..

[47] Bouhassira D., Moisset X., Jouet P., Duboc H., Coffin B., Sabate J.M. (2013). Changes in the modulation of spinal pain processing are related to severity in irritable bowel syndrome. Neurogastroenterol. Motil..

[48] Kim S.H., Lee Y., Lee S., Mun C.W. (2013). Evaluation of the effectiveness of pregabalin in alleviating pain associated with fibromyalgia: Using functional magnetic resonance imaging study. PLoS ONE.

[49] Jensen K.B., Kosek E., Petzke F., Carville S., Fransson P., Marcus H., Williams S.C., Choy E., Giesecke T., Mainguy Y. (2009). Evidence of dysfunctional pain inhibition in Fibromyalgia reflected in rACC during provoked pain. Pain.

[50] Russo A., Tessitore A., Esposito F., Marcuccio L., Giordano A., Conforti R., Truini A., Paccone A., d’Onofrio F., Tedeschi G. (2012). Pain processing in patients with migraine: An event-related fMRI study during trigeminal nociceptive stimulation. J. Neurol..

[51] Derbyshire S., Jones A., Devani P., Friston K.J., Feinmann C., Harris M., Pearce S., Watson J., Frackowiak R. (1994). Cerebral responses to pain in patients with atypical facial pain measured by positron emission tomography. J. Neurol. Neurosurg. Psychiatry.

[52] Kupers R., Lonsdale M.N., Aasvang E., Kehlet H. (2011). A positron emission tomography study of wind-up pain in chronic postherniotomy pain. Eur. J. Pain.

[53] Peyron R., Faillenot I., Pomares F.B., Le Bars D., Garcia-Larrea L., Laurent B. (2013). Mechanical allodynia in neuropathic pain. Where are the brain representations located? A positron emission tomography (PET) study. Eur. J. Pain.

[54] Apkarian A.V., Thomas P., Krauss B., Szeverenyi N. (2001). Prefrontal cortical hyperactivity in patients with sympathetically mediated chronic pain. Neurosci. Lett..

[55] Baliki M.N., Geha P.Y., Fields H.L., Apkarian A.V. (2010). Predicting value of pain and analgesia: Nucleus accumbens response to noxious stimuli changes in the presence of chronic pain. Neuron.

[56] Moana-Filho E.J., Bereiter D., Nixdorf D. (2015). Amplified Brain Processing of Dentoalveolar Pressure Stimulus in Persistent Dentoalveolar Pain Disorder Patients. J. Oral Facial Pain Headache.

[57] Hiramatsu T., Nakanishi K., Yoshimura S., Yoshino A., Adachi N., Okamoto Y., Yamawaki S., Ochi M. (2014). The dorsolateral prefrontal network is involved in pain perception in knee osteoarthritis patients. Neurosci. Lett..

[58] Dowdle L.T., Borckardt J.J., Back S.E., Morgan K., Adams D., Madan A., Balliet W., Hanlon C.A. (2019). Sensitized brain response to acute pain in patients using prescription opiates for chronic pain: A pilot study. Drug. Alcohol. Depend..

[59] Chen N., Zhang J., Wang P., Guo J., Zhou M., He L. (2015). Functional Alterations of Pain Processing Pathway in Migraine Patients with Cutaneous Allodynia. Pain Med..

[60] Russo A., Esposito F., Conte F., Fratello M., Caiazzo G., Marcuccio L., Giordano A., Tedeschi G., Tessitore A. (2017). Functional interictal changes of pain processing in migraine with ictal cutaneous allodynia. Cephalalgia.

[61] Russo A., Tessitore A., Silvestro M., Di Nardo F., Trojsi F., Del Santo T., De Micco R., Esposito F., Tedeschi G. (2019). Advanced visual network and cerebellar hyperresponsiveness to trigeminal nociception in migraine with aura. J. Headache Pain.

[62] Schreiber K.L., Loggia M.L., Kim J., Cahalan C.M., Napadow V., Edwards R.R. (2017). Painful After-Sensations in Fibromyalgia are Linked to Catastrophizing and Differences in Brain Response in the Medial Temporal Lobe. J. Pain.

[63] Boland E.G., Selvarajah D., Hunter M., Ezaydi Y., Tesfaye S., Ahmedzai S.H., Snowden J.A., Wilkinson I.D. (2014). Central pain processing in chronic chemotherapy-induced peripheral neuropathy: A functional magnetic resonance imaging study. PLoS ONE.

[64] Kashima H., Ikemura T., Hayashi N. (2013). Regional differences in facial skin blood flow responses to the cold pressor and static handgrip tests. Eur. J. Appl. Physiol..

[65] Young I.A., Dunning J., Butts R., Cleland J.A., Fernandez-de-Las-Penas C. (2019). Psychometric properties of the Numeric Pain Rating Scale and Neck Disability Index in patients with cervicogenic headache. Cephalalgia.

[66] Young I.A., Dunning J., Butts R., Mourad F., Cleland J. (2019). Reliability, construct validity, and responsiveness of the neck disability index and numeric pain rating scale in patients with mechanical neck pain without upper extremity symptoms. Physiother Theory Pr..

[67] Kocsis L., Herman P., Eke A. (2006). The modified Beer-Lambert law revisited. Phys. Med. Biol..

[68] Scholkmann F., Wolf M. (2013). General equation for the differential pathlength factor of the frontal human head depending on wavelength and age. J. Biomed. Opt..

[69] Klein F., Kranczioch C. (2019). Signal Processing in fNIRS: A Case for the Removal of Systemic Activity for Single Trial Data. Front. Hum. Neurosci..

[70] Saager R.B., Berger A.J. (2005). Direct characterization and removal of interfering absorption trends in two-layer turbid media. J. Opt. Soc. Am. A Opt. Image Sci. Vis..

[71] Stute K., Hudl N., Stojan R., Voelcker-Rehage C. (2020). Shedding Light on the Effects of Moderate Acute Exercise on Working Memory Performance in Healthy Older Adults: An fNIRS Study. Brain Sci..

[72] Meissel K., Yao E. (2024). Using Cliff’s Delta as a Non-Parametric Effect Size Measure: An Accessible Web App and R Tutorial. Pract. Assess. Res. Eval..

[73] Pourshoghi A., Zakeri I., Pourrezaei K. (2016). Application of functional data analysis in classification and clustering of functional near-infrared spectroscopy signal in response to noxious stimuli. J. Biomed. Opt..

[74] Geissler C.F., Frings C., Domes G. (2025). The effects of stress on working-memory-related prefrontal processing: An fNIRS study. Stress.

[75] Kalia V., Vishwanath K., Knauft K., Vellen B.V., Luebbe A., Williams A. (2018). Acute Stress Attenuates Cognitive Flexibility in Males Only: An fNIRS Examination. Front. Psychol..

[76] Ma L., Xu K., Ding J., Gao J., Wang X. (2022). Physical Stress Attenuates Cognitive Inhibition: An fNIRS Examination. Adv. Exp. Med. Biol..

[77] Meier J.K., Schwabe L. (2024). Consistently increased dorsolateral prefrontal cortex activity during the exposure to acute stressors. Cereb. Cortex..

[78] Hazra S., Venkataraman S., Handa G., Yadav S.L., Wadhwa S., Singh U., Kochhar K.P., Deepak K.K., Sarkar K. (2020). A Cross-Sectional Study on Central Sensitization and Autonomic Changes in Fibromyalgia. Front. Neurosci..

[79] Rosenbaum D., Int-Veen I., Laicher H., Torka F., Kroczek A., Rubel J., Lawyer G., Bürger Z., Bihlmaier I., Storchak H. (2021). Insights from a laboratory and naturalistic investigation on stress, rumination and frontal brain functioning in MDD: An fNIRS study. Neurobiol. Stress.

[80] Pistoia F., Salfi F., Saporito G., Ornello R., Frattale I., D’Aurizio G., Tempesta D., Ferrara M., Sacco S. (2022). Behavioral and psychological factors in individuals with migraine without psychiatric comorbidities. J. Headache Pain.

[81] Scaini S., Davies S., De Francesco S., Pelucchi A., Rubino S., Battaglia M. (2025). Altered pain perception and nociceptive thresholds in major depression and anxiety disorders: A meta-analysis. Neurosci. Biobehav. Rev..

[82] Othman R., Jayakaran P., Swain N., Dassanayake S., Tumilty S., Mani R. (2021). Relationships Between Psychological, Sleep, and Physical Activity Measures and Somatosensory Function in People With Peripheral Joint Pain: A Systematic Review and Meta-Analysis. Pain Pract..

[83] Carneiro A.M., Pacheco-Barrios K., Andrade M.F., Martinez-Magallanes D., Pichardo E., Caumo W., Fregni F. (2024). Psychological Factors Modulate Quantitative Sensory Testing Measures in Fibromyalgia Patients: A Systematic Review and Meta-Regression Analysis. Psychosom. Med..

[84] Emmert K., Breimhorst M., Bauermann T., Birklein F., Rebhorn C., Van De Ville D., Haller S. (2017). Active pain coping is associated with the response in real-time fMRI neurofeedback during pain. Brain Imaging Behav..

[85] Seminowicz D.A., Davis K.D. (2006). Cortical responses to pain in healthy individuals depends on pain catastrophizing. Pain.

[86] Paulesu E., Sambugaro E., Torti T., Danelli L., Ferri F., Scialfa G., Sberna M., Ruggiero G.M., Bottini G., Sassaroli S. (2010). Neural correlates of worry in generalized anxiety disorder and in normal controls: A functional MRI study. Psychol. Med..

[87] Monkul E.S., Silva L.A., Narayana S., Peluso M.A., Zamarripa F., Nery F.G., Najt P., Li J., Lancaster J.L., Fox P.T. (2012). Abnormal resting state corticolimbic blood flow in depressed unmedicated patients with major depression: A (15)O-H(2)O PET study. Hum. Brain Mapp..

[88] Wang H.Y., Zhang X.X., Si C.P., Xu Y., Liu Q., Bian H.T., Zhang B.W., Li X.L., Yan Z.R. (2018). Prefrontoparietal dysfunction during emotion regulation in anxiety disorder: A meta-analysis of functional magnetic resonance imaging studies. Neuropsychiatr. Dis. Treat.

[89] Waxenbaum J.A., Reddy V., Varacallo M.A. (2025). Anatomy, Autonomic Nervous System.

[90] Drummond P.D. (2006). Immersion of the hand in ice water releases adrenergic vasoconstrictor tone in the ipsilateral temple. Auton. Neurosci..

[91] Saad M., Gabr W., El-Azouni O., Enein A.F. (2019). Cardiovascular sympathetic nervous system response to cold pressor test among patients with migraine. Med. Sci..

[92] Drummond P.D., Granston A. (2003). Facilitation of extracranial vasodilatation to limb pain in migraine sufferers. Neurology.

[93] Zhang L., Qiu S., Zhao C., Wang P., Yu S. (2021). Heart Rate Variability Analysis in Episodic Migraine: A Cross-Sectional Study. Front. Neurol..

[94] Saboo N., Kacker S., Sorout J. (2025). Effects of Cognitive Behavior Therapy on Heart Rate Variability among Medical Students at a Teaching Institution in Jaipur, India. APIK J. Intern. Med..

[95] Amra B., Ghadiry F., Vaezi A., Nematollahy A., Radfar N., Haghjoo S., Penzel T., Morin C.M. (2023). Effect of one-shot cognitive behavioral therapy on insomnia and heart rate variability of health care workers at the time of COVID-19 pandemic: A randomized controlled trial. Sleep Breath.

[96] Prados G., Miro E., Martinez M.P., Sanchez A.I., Pichot V., Medina-Casado M., Chouchou F. (2022). Effect of Cognitive-Behavioral Therapy on Nocturnal Autonomic Activity in Patients with Fibromyalgia: A Preliminary Study. Brain Sci..

[97] McGeary D.D., Resick P.A., Penzien D.B., McGeary C.A., Houle T.T., Eapen B.C., Jaramillo C.A., Nabity P.S., Reed D.E., Moring J.C. (2022). Cognitive Behavioral Therapy for Veterans With Comorbid Posttraumatic Headache and Posttraumatic Stress Disorder Symptoms: A Randomized Clinical Trial. JAMA Neurol..

[98] Ong W.Y., Stohler C.S., Herr D.R. (2019). Role of the Prefrontal Cortex in Pain Processing. Mol. Neurobiol..

[99] Hejl C., Burns C., Cherry J., Bradford A., Szabo Y.Z. (2023). Understanding Differences Between Veterans and Civilians on a Range of Biopsychological Domains: Descriptive Report from the MIDUS II Study. J. Veterans Stud..

[100] Bishop S.A., Dech R.T., Guzik P., Neary J.P. (2018). Heart rate variability and implication for sport concussion. Clin. Physiol. Funct. Imaging.

[101] Iser C., Arca K. (2022). Headache and Autonomic Dysfunction: A Review. Curr. Neurol. Neurosci. Rep..

[102] Carlson B.E., Arciero J.C., Secomb T.W. (2008). Theoretical model of blood flow autoregulation: Roles of myogenic, shear-dependent, and metabolic responses. Am. J. Physiol. Heart Circ. Physiol..

[103] Johnson P. (1986). Brief Review Autoregulation of Blood Flow. Circ. Res..

[104] Chu M., Nguyen T., Pandey V., Zhou Y., Pham H.N., Bar-Yoseph R., Radom-Aizik S., Jain R., Cooper D.M., Khine M. (2019). Respiration rate and volume measurements using wearable strain sensors. NPJ Digit. Med..

[105] Fuller D., Colwell E., Low J., Orychock K., Tobin M.A., Simango B., Buote R., Van Heerden D., Luan H., Cullen K. (2020). Reliability and Validity of Commercially Available Wearable Devices for Measuring Steps, Energy Expenditure, and Heart Rate: Systematic Review. JMIR mHealth uHealth.

[106] Kumar S., Yadav S., Kumar A. (2024). Blood pressure measurement techniques, standards, technologies, and the latest futuristic wearable cuff-less know-how. Sens. Diagn..

[107] Damoun N., Amekran Y., Taiek N., Hangouche A.J.E. (2024). Heart rate variability measurement and influencing factors: Towards the standardization of methodology. Glob Cardiol. Sci. Pract..

[108] Li S., Sung B., Lin Y., Mitas O. (2022). Electrodermal activity measure: A methodological review. Ann. Tour. Res..

[109] Dawson M., Schell A., Filion D., Berntson G., Quigley K., Lozano D., Lorig T., Berntson G. (2007). Autonomic and Somatic Nervous System. The Handbook of Psychophysiology.

[110] Lorenz J., Minoshima S., Casey K.L. (2003). Keeping pain out of mind: The role of the dorsolateral prefrontal cortex in pain modulation. Brain.

[111] Stankewitz A., Mayr A., Irving S., Witkovsky V., Schulz E. (2023). Pain and the emotional brain: Pain-related cortical processes are better reflected by affective evaluation than by cognitive evaluation. Sci. Rep..

[112] Peng K., Steele S.C., Becerra L., Borsook D. (2018). Brodmann area 10: Collating, integrating and high level processing of nociception and pain. Prog. Neurobiol..

[113] Tsuzuki D., Dan I. (2014). Spatial registration for functional near-infrared spectroscopy: From channel position on the scalp to cortical location in individual and group analyses. Neuroimage.

[114] Seminowicz D.A., Moayedi M. (2017). The Dorsolateral Prefrontal Cortex in Acute and Chronic Pain. J. Pain.

[115] Borsook D., Edwards R., Elman I., Becerra L., Levine J. (2013). Pain and analgesia: The value of salience circuits. Prog. Neurobiol..

[116] Alshelh Z., Marciszewski K.K., Akhter R., Di Pietro F., Mills E.P., Vickers E.R., Peck C.C., Murray G.M., Henderson L.A. (2018). Disruption of default mode network dynamics in acute and chronic pain states. Neuroimage Clin..

[117] Kong J., Jensen K., Loiotile R., Cheetham A., Wey H.Y., Tan Y., Rosen B., Smoller J.W., Kaptchuk T.J., Gollub R.L. (2013). Functional connectivity of the frontoparietal network predicts cognitive modulation of pain. Pain.

[118] De Ridder D., Vanneste S., Smith M., Adhia D. (2022). Pain and the Triple Network Model. Front. Neurol..

